# Self-reported Provision of Preconception Care and Associated Factors

**DOI:** 10.24248/eahrj.v8i1.752

**Published:** 2024-03-28

**Authors:** Everlyne N Morema, Collins Ouma, Robert Egessa, Lydia Nyachiro, Morris Shisanya

**Affiliations:** a School of Nursing Midwifery and Paramedic sciences, Masinde Muliro University of Science and Technology, Kenya; b School of Public Health and Community Development, Maseno University, Kenya; c School of Nursing, Kibabii University, Kenya

## Abstract

**Background:**

Preconception care (PCC) is the provision of health interventions to women and couples before conception occurs and is valuable in promoting healthy maternal, birth, and neonatal health outcomes. In Africa, more so in Kenya, maternal and neonatal health indicators have remained poor. The key constraint limiting progress is the gap between what is needed and what exists in terms of skills and availability of human resources & infrastructures in the face of increased demand. This gap was yet to be measured for PCC in Kenya, more so in Kisumu County.

**Methods:**

Using a cross-sectional design, this study specifically sought to determine the rate of self-reported PCC provision and to illustrate how it is influenced by health provider characteristics. Structured interviews were conducted with health providers (n=476) to ascertain their knowledge, perceptions and practice of PCC care. The significance of the differences in means was determined by the Student's t test and linear regression were used to show the relationship between the health provider characteristics and the PCC provision rate.

**Results:**

Self-reported PCC provision was estimated at 39%. There was a significant difference in the mean for cadres {nurses (M=70.04, SD=8.951) and non-nurses (M=71.90, SD=8.732); t (473) =-2.23, *P=.026*)}, years of experience up to 5 years (M=72.04, SD=8.417) and more than 5 years (M=69.89, SD=9.283); t (465) =2.63, *P=.009*, the mean provision per level (M=60.21, SD=4.902; t (26)=-5.06, *P<.001*) and type of service (M=69.36, SD=4.924; t (26) =4.63, *P<.001*). A significant regression model was found, and the model statistics were F (2,464) =5.97, *P=.003*, R2=.03. Only cadre (b=0.01, t (464) =2.23, *P=.026*) and years of experience (b=-0.13, t (464) =-2.79, *P=.005*) were significant determinants of PCC provision. The health workers felt PCC was an important service whose provision was low due to inadequate human capital investment.

**Conclusion:**

Self-reported provision of PCC by health workers was relatively low and was influenced by the cadre of health workers and their years of experience. It specifically demonstrated the importance of various aspects of human capital, i.e., knowledge, perceptions, competence and adequacy of training in the provision of this care. Furthermore, it showed that the nursing cadre has a higher probability of providing this care. Investing in on-the-job training for health providers, especially nurses, and providing care in primary health facilities in rural areas can improve PCC service delivery.

## INTRODUCTION

Preconception Care (PCC) is the provision of biomedical, behavioral and social health interventions to people considering pregnancy before conception occurs.^1^ It aims at improving their health status, reducing behaviors and individual factors as well as environmental factors that contribute to poor maternal and child health outcomes.^2^ In the current study, preconception care was conceptualized to include interventions to be provided to women of childbearing age in the period before pregnancy (at least 1 year) or between consecutive pregnancies. PCC is an important component of the continuum of maternal health, therefore plays a big role in improving maternal and, neonatal health indicators.^3^ PCC is part of the recommended national policy framework for WHO member countries and is recognized as an important contributor to non-communicable disease prevention and control.^2,4^

PCC has a range of benefits that include reduction of maternal and child mortality, prevention of unintended pregnancies, reduced complications during pregnancy and delivery, prevention of stillbirths, preterm birth and low birth weight, birth defects, neonatal infections, underweight and stunting, prevention of vertical transmission of HIV and other sexually transmitted infections (STIs), it lowers the risk of some forms of childhood cancers, including reducing the risk of type 2 diabetes and cardiovascular disease later in life.^5^ Thus, PCC is key in improving maternal and neonatal health indicators and has shown to create demand for antenatal care services.^6^ Yet, most countries in Sub-Saharan Africa use a model of maternal new born care that puts less emphasis on this service.^7,8^ PCC is lumped together with family planning with efforts to improve family planning uptake overshadowing the implementation of PCC. Vital fetal development occurs in the first weeks of pregnancy often before the mother realizes she is pregnant and is greatly affected by environmental and physiological factors affecting the mother immediately before the period.^1^ There is need to reposition it to create synergy with the efforts being put in promoting ANC and postnatal care for the achievement of the sustainable development agenda.

The UN Assembly of 2015 declared the 3^rd^ Sustainable Development Goal to ensure healthy lives and promote wellbeing for all ages.^9^ The maternal and neonatal mortality rates are still high globally but are worse in Sub Saharan Africa.^10^ In 2020, the maternal mortality ratio in the African Region was estimated at 531 deaths per 100 000 live births.^11^ The challenge in achieving the desired maternal and neonatal health has been cited as a gap between what is needed and what exists in terms of skills and supplies, among other resources, in the face of increased demand.^12^ This gap has yet to be ascertained in the case of preconception care. Consequently, this could have led to an imbalance in the continuum of care, with priority being given to interventions targeting prenatal, perinatal and postpartum periods at the expense of preconception care, as evidenced in the national roadmap to the attainment of MNH's strategic objective 5.^13^

The uptake of PCC is low worldwide and worse in low-income countries since it is not a widespread concept as of yet.^6^ The rate of use of these services was found to be between 18.7% and 45% among diabetes patients in Canada.^14^ Another study in England estimated it at 45%,^15^ while in United States of America, two studies estimated it at 33% and 47.7%.^16,17^ Studies have shown that despite being offered this service very few couples agree to be recruited to the care.^6^ The studies in Canada, USA and England showed that socio-economic status, previous pregnancies and age influenced the uptake of this particular service.^14,15,17^ Pooled prevalence of utilization of PCC in Africa has been estimated at 18.72%.^18^ A number of individual studies conducted in SSA region have shown inconsistency in the utilization of PCC among women in the reproductive age group with prevalence rates ranging from 13.4% to 34.1%.^10^ In Kenya, uptake of PCC was estimated at 25.8% which is suboptimal and significantly lower than that of developed countries.^19^

There is scanty literature on rate of provision of this service which relates to the level of implementation and is essential to understand the accessibility of this service. Inadequate implementation of comprehensive PCC has been cited as a barrier to its uptake.^5^ This information gap can frustrate efforts aimed at increasing its utilization/uptake.

The WHO identified improvement of health systems (infrastructure, management, distribution of goods, training of providers) to deliver preconception care as a priority research area. Preconception care is key in improving maternal and neonatal health indicators.^6^ It also creates demand for other services, such as antenatal care. However, the PCC rate of provision has not been measured in the current setting. Health care service provision depends on factors such as providers' experiences, individualized ability and personalities.^20^

Thus, the various cadres of health care professionals necessary for its implementation diversify preconception care. These include nurses, clinical officers, nutritionists and community health extension workers as well as community health assistants. The training of these professionals differs greatly in terms of curriculum and length of training. The training levels also differ; that is, we have those trained at the certificate, diploma, undergraduate and postgraduate levels. This raised the question of whether these disparities affect preconception care provision. It further questioned the adequacy of training for the role of implementing this care and whether some cadres have a competitive advantage of intellectual capital required in PCC provision. This study, therefore, estimated the rate of preconception care provision and determined how it is influenced by health worker characteristics in health facilities in Kisumu County of Kenya. The findings of this study may guide stakeholders in program planning for the integration of preconception care with other services in the continuum of maternal and newborn health.

## METHODS

### Study Area and Design

An analytical, cross-sectional study was carried out at facilities in Kisumu County. Kisumu County is one of the newly devolved counties of Kenya. It covers an area of 805 square miles and has a population of 1,155,574. It has 7 sub-counties, namely, Kisumu West, Kisumu Central, Kisumu East, Seme, Muhoroni, Nyando and Nyakach. Kisumu County has a total of 129 public health facilities. The risk factors for unhealthy pregnancy that are amenable to PCC in Sub-Saharan Africa are present in Kenya and are more pronounced in Kisumu County as per previous Kenya Demographic Health Surveys (KDHS).^6,20^ According to these surveys, the median age at first birth is lowest in Nyanza at 18.7 years; birth interval of less than 18 months is common in Nyanza Region at 7.6 per 1000 live births, second only to North Eastern.

Kisumu County, which is situated in Nyanza region is at the lead at 8.8. Success in achieving reproductive intention is lowest in Nyanza. The gap between wanted and unwanted fertility is widest in Nyanza at 1.5 in as compared to the national gap which shows women having 0.6 children more than the number they intend to get. The proportion of reproductive aged women who are overweight has increased significantly from 25-33%'since 2008.^6,20^

### Sample Size and Sampling

The sample size of n=28 was derived from a sampling frame of 129 health facilities using the corrected Fishers formula.^21^



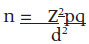



Where,

n = the estimated number of clients to be interviewed

Z = confidence interval width at 95% level which is equivalent to 1.96

p = proportion of the characteristic under study. (The proportion of the study population who offer preconception care, p=43 rate from a previous study)

q = 1-p

d = is the error term which is estimated at .05

Therefore,



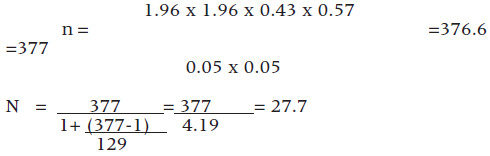



Thus, a sample size of n=28 health facilities arrived at.

Further, the minimum sample size for the health workers was derived in similar manner where the population of health workers was 1318 (County health records).



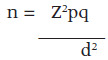



Where,

n = the estimated number of health workers to be interviewed

Z = confidence interval width at 95% level which is equivalent to 1.96

p=proportion of the characteristic under study (The proportion of the study population who offer preconception care, p=43 rate from a previous study):

q = 1-p

d = is the error term which is estimated at 0.05,

Therefore,



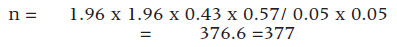



Since the health provider population was a finite population, the formula was corrected as follows:



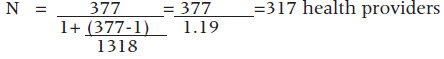



To ensure reliability of the results, simple random sampling is recommended and if not, a sample size twice as would be needed under simple random sampling should be used. Previous studies have recommended the use of a design effect factor (DEFF) of 1.5 as a benchmark in respondent-driven sampling.^22^ This study employed stratified and purposive sampling, and to reduce sampling error, the calculated sample sizes of n=317 were multiplied by 1.5 to arrive at n=476.

The study employed multistage sampling. First, stratified sampling was performed, whereby the 7 sub-counties served as strata. Purposive sampling was used to select 2 high-volume dispensaries, health centres, and sub-county hospitals from each subcounty. To arrive at the health provider sample, proportionate sampling of the cadres was done based on the number of health workers in each cadre in the county.

### Data Collection

Quantitative data were collected with the aid of a self-administered questionnaire. The respondents were reproductive health service providers within the facilities under study. They were drawn from departments that provided antenatal care, postnatal care, well-child health services, gynaecological care and childbirth services. This questionnaire was adopted from a previous study in Ethiopia as alluded to earlier.^23^ The variables that were elicited as depicted in the conceptual framework were level of training, knowledge of PCC for each cadre, perceptions of PCC, and adequacy of training on PCC.

The level of training was elucidated by a prompt in the questionnaire. In the questionnaire, the health care providers were asked to list at least 5 PCC interventions. Level of knowledge was assessed against the 5 responses, with the highest being 5 correct responses and the lowest being 0-1 correct response. The attitude was measured using a Likert scale where the health workers were asked questions on their perception of the importance of preconception and their perceived competence on at least 5 interventions. The Likert scale had 5 options ranging from strongly agree to strongly disagree.

### Data Management and Statistical Analysis

The self-administered questionnaires were checked for completeness. Data forms were created with Epi Info version 7,^24^ verified and cleaned. It was then exported to SPSS version 21.0, where both descriptive and inferential analyses were performed.^25^ Proportions of the *yes* and *no* responses on whether each of the interventions for each service in the package as per the checklist was provided were calculated and presented as percentages. Then, the means for all of the services in the package were determined. The significance of the difference in the means was determined by the one sample T test at a *P* value of equal to or less than 0.05. Furthermore, the rate of use was derived from the proportion of women that each health provider indicated they had given care.

Descriptive statistics are presented in contingency tables with counts and proportions. Bivariate analysis was performed on the level of knowledge and practice. The differences in proportions of the other variables were determined by the chi-square statistic. Variables with significant *P* values were fitted into a multilinear logistic regression analysis to adjust for confounders and thus determine their influence on the provision of PCC. The strength of the association was measured by the odds ratio and 95% confidence interval. Furthermore, the independent sample T test was used to show the significance in the differences in means for the various characteristics of the health care providers.

## RESULTS

A total of 28 facilities were involved in the study, with 50% (14) of the facilities being inpatient service providers and the rest being both inpatient and outpatient service providers. Most of the facilities (64.3%, 18) were in rural settings, while the remaining 35.7% (10) came from urban settings.

A total of 475 reproductive health workers responded to the questionnaire out of the sample of n=476. The resulting response rate was 99.8%.

### Demographics

Table 1 presents the demographic characteristics of the health care provider respondents. The mean PCC engagements reported were 3.7 for every 10 clients seen, which provided a basis for stratification. When stratified into those who reported having had ≤3 PCC engagements for every 10 clients seen verses those who had >3, there was a significant difference between the cadres and facility levels.

**TABLE 1:** Demographic Characteristics of Health Providers and Number of PCC Engagements

Characteristics	Grouping	Preconception engagements		Total	*P value*
		≤3	≥3		
Age (Years)	≤30	46 (106)	54 (125)	49 (231)	.612
	Above 30	44 (105)	56 (136)	51 (241)	
	Total	45 (211)	55 (261)	100 (472)	
Marital status	Married	44 (143)	56 (180)	69 (325)	.749
	Not Married	46 (67)	54 (80)	31 (147)	
	Total	44 (210)	54 (260)	100 (472)	
Sex	Male	46 (59)	54 (69)	27 (128)	.716
	Female	44 (153)	56 (193)	73 (346)	
	Total	45 (212)	55 (262)	100 (474)	
Cadre	Nurse	46 (123)	54 (142)	56 (265)	.013
	Doctor	21 (8)	79 (30)	8 (38)	
	C.O	53 (40)	47 (35)	16 (75)	
	Nutrition	48 (23)	52 (25)	10 (48)	
	Lab Tech	37 (18)	63 (31)	10 (49)	
	Total	45 (212)	55 (263)	100 (475)	
Level of Education	Certificate	43 (3)	4	1 (7)	.867
	Diploma	46 (139)	165	64 (304)	
	Degree	43 (69)	90	33 (159)	
	Masters	20 (1)	4	1 (5)	
	Total	45 (212)	263	100 (475)	
Experience	≤5 years	47 (112)	53 (127)	51 (239)	.400
	>5 Years	43 (98)	57 (130)	49 (228)	
	Total	45 (210)	55 (257)	100 (467)	
Facility Level	Level 2 & 3	52 (58)	48 (54)	24 (112)	.049
	Level 4 &5	42 (152)	58 (207)	76 (359)	
	Total	45 (210)	55 (261)	100 (471)	

Data are in proportions, counts in brackets. C. O-clinical officer. Statistical significance was determined by x2 at P≤0.05. K- Kisumu

### The Influence of Human Capital on the Provision of Preconception Care

Human capital is the knowledge, skills and experiences owned and used by individuals. In this study, it was conceptualized as knowledge of preconception care for each cadre of service providers, perceptions of the importance of preconception care, their practice in the implementation of this care and adequacy of training on preconception care.

### Knowledge

Those workers who were able to give 5 correct interventions in the preconception package were considered knowledgeable. From the study, at least 50% of the health workers were found to be knowledgeable about preconception care, with the exception of lab technicians. The highest proportion of health workers found knowledgeable at 76.3% (n=38) were doctors. The knowledge status varied across the various health provider characteristics. There was a significant difference in knowledge among the cadres (*P*=.023) and the level of education (*P*=.01) (Table 2).

**TABLE 2:** Health Worker Characteristics and Knowledge Status

Characteristics	Grouping	Knowledgeable		Total	*P value*
		Yes	No		
Age	30 and Below	106	125	231	.612
	Above 30	105	136	241	
	Total	211	261	472	
Marital status	Married	143	180	325	.749
	Not Married	67	80	147	
	Total	210	260	472	
Sex	Male	75	53	128	.408
	Female	188	158	346	
	Total	263	211	474	
Cadre	Nurse	138	127	265	.023
	Doctor	29	9	38	
	Clinical Officer	46	29	75	
	Nutrition	28	20	48	
	Lab Tech	22	27	49	
	Total	263	212	475	
Level of Education	Degree and above	104	60	164	.01
	Diploma or Certificate	159	152	311	
	Total	263	212	475	
Experience	Up to 5	138	101	239	.267
	More than 5 Years	120	108	228	
	Total	258	209	467	
Facility Level	Level 2 & 3	61	51	112	.618
	Level 4 & 5	200	158	358	
	Total	261	209	470	

Significance of the differences in proportions was determined by the x2 at *P=.05*

### Perceptions of preconception care.

Regarding perceptions, 86.4% (n=472) of the staff appreciate that preconception care is as important as other health packages, such as ANC, in the continuum of maternal health care. They also believe that a hospital setting is adequate to provide care (67.4%, n=472) and feel that PCC should be a priority in their daily workload (49.5%, n=472). Many note that there is inadequate time to provide PCC (41%, n=472), while a good number (22%, n=472) are unsure.

A total of 81.6% of the doctors interviewed expressed confidence in their ability to provide comprehensive PCC, while only 29.9% and 20.8% of the nurses and lab technicians felt competent, respectively. Consequently, these perceptions were found to have a significant association with the provision of preconception care. There was a significant difference in the means for those who felt PCC was important (*P*=.003), was a priority (*P*=.048) and the hospital setting was the best place to offer it (*P*=.007) (Table 3).

**TABLE 3:** Perceptions and PCC

Characteristic	Grouping	N	M	SD	SEM	t	Df	*P*
Preconception care is as important as ANC	Strongly Agree	297	70.51	8.409	0.488	2.96	345	.003
	Strongly Disagree	50	74.34	8.775	1.241			
A hospital setting is the best place to provide preconception care	Strongly Agree	184	68.01	8.496	0.626	2.01	73.4	.048
	Strongly Disagree	51	70.98	9.547	1.337			
PCC is a high priority in my workload	Strongly Agree	111	68.49	8.695	0.825	2.75	141	.007
	Strongly Disagree	32	73.38	9.435	1.668			
No enough time to provide PCC	Strongly Agree	105	71.98	8.878	0.866	−1.41	191	.159
	Strongly Disagree	88	70.15	9.1	0.97			
I do not have the appropriate skills to offer preconception care	Strongly Agree	116	73.75	8.236	0.765	−2.35	259	.020
	Strongly Disagree	145	71.13	9.503	0.789			

Statistical significance was determined by Student's t test at *P≤.05.*

N=sample size

M=mean SD=standard deviation

SEM=standard error of the mean

t=test statistic

Df=Degrees of freedom

P=level of significance

Most doctors, 65.6% (n=38), felt their training was adequate to prepare them to give care, while only 29.2% of the laboratory technicians had similar sentiments. Furthermore, the respondents were required to indicate whether they had ever learned about specific services in the PCC package and their responses documented. A total of 40.3% (n=471) of health providers indicated that they were trained on the risks associated with tobacco use and how to discourage it, while only 1.1% (n=471) felt they were adequately prepared to manage and prevent sexually transmitted infections as part of PCC. The outcomes deduce that the health providers were exposed to most of the concepts on PCC during their preservice training. However, a good number indicated that they had not learnt about vaccine-preventable infections of preconception care relevance N=189 (40.8%), tobacco use and its relevance in preconception care 190 (40.3%) and female genital mutilation diagnosis and management 159 (33.5%).

### Human capital characteristics and PCC provision.

An independent samples t-test was conducted to compare the provision of PCC and various characteristics of the health care providers. There was a significant difference in the means of reported provision of PCC between the cadres for nurses (M=70.04, SD=8.951) and other providers (M=71.90, SD=8.732); t (473) =-2.23, *P*=.026, years of experience up to 5 years (M=72.04, SD=8.417) and more than five years (M=69.89, SD=9.283); t(465)= 2.63 *P*=.009 (Table 4). Although the t test did not show a significant difference in the mean of provision as per knowledge status, the lack of it was cited as a challenge in the provision of PCC during the FGD. As was the case for cadre of health workers affecting provision of PCC.

**TABLE 4:** Human Capital and Preconception Care Provision

Characteristic	Grouping	N	M	SD	SEM	t	df	*P*
Age	30 and below	231	71.51	8.721	0.574	1.45	470	.148
	Above 30	241	70.32	9.053	0.583			
Sex	Male	128	70.4	9.67	0.855	−0.63	205.59	.531
	Female	346	71.01	8.594	0.462			
Marital status	Married	325	70.98	9.055	0.502	0.56	470	.579
	Not Married	147	70.49	8.556	0.706			
Level of Education	Degree and above	164	71.79	8.687	0.678	1.64	473	.101
	Diploma or Certificate	311	70.38	8.977	0.509			
Cadre	Nurses	265	70.04	8.951	0.55	−2.28	473	.023
	Non Nurses	210	71.9	8.732	0.603			
Experience (Years)	Up to 5	239	72.04	8.417	0.544	2.63	465	.009
	More than 5	228	69.89	9.283	0.615			
Adequate PCC training	Yes	222	71.5	8.924	0.599	1.46	473	.145
	No	253	70.31	8.847	0.556			
Knowledgeable on PCC	Yes	263	71.08	8.654	0.534	0.6	473	.552
	No	212	70.59	9.196	0.632			
Competent	Yes	160	71.3	9.111	0.72	0.76	473	.448
	No	315	70.64	8.788	0.495			

Statistical significance was determined by Student's t test at *P≤.05.*

N=sample size

M=mean

SD=standard deviation

A linear regression analysis model was applied to adjust for confounders in the original t test and multilinear logistic regression and significant determinants of PCC service provision. The model applied allows the entry of method and cases to be excluded listwise. ANOVA was used to determine model significance. A significant regression model was found, and the model statistics were F (2,464) =5.97, *P*=.003, R^2^=.03. Only cadre (b=0.01, t (464) =2.23, *P*=.026) and years of experience (b=-0.13, t (464) =-2.79, *P*=.005) were significant determinants of PCC provision.

## DISCUSSION

From the study, it was realized that health workers reported suboptimal PCC engagements yet health providers were found to be considerably more knowledgeable about preconception care than in other studies done in Africa.^7,23,26–28^ This finding collaborated with the focus group discussions, which demonstrated that the health workers had a good grasp of what PCC was. A study in Egypt reported a knowledge level of 22% for all health workers, while another study in Ethiopia estimated it at 31%.^29,30^ The latter study further revealed that health workers who reported access to the internet were significantly more knowledgeable than others. Thus, the difference could be attributed to the fact that the questionnaires for this study were self-administered and there was a possibility of access to information from the internet via mobile technology. Nevertheless, the highest number of health workers who were found knowledgeable were doctors. This is congruent with a study in Taiwan that found the level of knowledge of general practitioner, a cadre similar to doctors.^31^ This high level of knowledge in this cadre could be attributed to the length of training and consequently the depth at which the curriculum delved into preconception information.

The level of knowledge was higher among those providers from referral facilities than those in primary level facilities (level 2 and 3). This is in agreement with another study, which demonstrated that the knowledge level was higher among those workers working in larger facilities.^30^ Furthermore, the staff indicated that they had never had any formal updates on preconception care, and they also had varied views about the adequacy of the training received at their training institutions. This level of knowledge and its felt adequacy could be related to the number of years of training for this cadre of health workers, implying the depth of interaction with preconception care content. Thus, to bridge this gap in knowledge within the health worker population, in-service training programs or updates are necessary. Regarding perceptions, preconception care was viewed as an important as other health packages, such as ANC, in the continuum of maternal health care. This is different and favourable considering other studies, such as Mazza et al 2013, where health workers indicated that preconception care was the lowest priority in their daily workloads.^32^ The health workers also believed that a hospital setting is adequate to provide care and felt that PCC should be a priority in their daily workload. The study demonstrated that these perceptions significantly influenced the provision of preconception care.

Many noted that there was inadequate time to provide PCC, while a good number were unsure. A lack of enthusiasm on the part of health workers was identified as one of the major barriers to the provision of preconception care.^5,12,13^ This lack of enthusiasm could be related to inadequate rewards for effort, a heavy workload that puts pressure on time resources and an unsupportive environment. This was also suggested by a study that demonstrated that health providers who earned higher pay were likely to provide PCC.^30^ Doctors expressed confidence in their ability to provide comprehensive PCC, yet nurses and lab technicians felt less competent. Furthermore, the study was able to demonstrate that the felt incompetency to provide this care negatively influenced its provision. This may be a true finding considering that health workers do not practice what they do not know. Knowledge is an important predictor for the implementation of preconception care since doctors who were found highly knowledgeable and felt competent to provide care ultimately reported higher number of PCC engagements.^30^ In the study in Ethiopia, those with poor PCC knowledge had 4 times higher odds of not practicing PCC. Investing in human capital through in-service training is important. This is because health workers are primarily responsible for putting up-to-date evidence into practice, preconception care included.^30^

The results revealed that the cadres of health workers influence the provision of PCC. Specifically, our results suggested that nurses were less likely to provide care. This is in keeping with another study that concluded that nurses and midwives had 2 times higher odds of not providing preconception care. This may be because nurses are found in all levels of facilities and form a larger proportion of health workers. Strategies to increase the provision of PCC targeting nurses may have the greatest impact. This study was able to demonstrate that the work experience of health workers negatively influenced the provision of PCC. The mean provision of PCC was found to be higher in among those with less than 5 years of experience than that of those who had worked for more than 5 years. This is unusual since it is appreciated that clinical experience increases prowess in practice. However, preconception is a relatively new concept that may not have been included in the curriculum for those who underwent their basic training in the earlier years. Thus, there is a need for on the job training or updates on current reproductive health issues, including PCC.

## CONCLUSIONS

The results show a low rate of self-reported PCC provision at 39%. Human capital investment in preconception care is low in the study setting. The results demonstrate that human capital is a determinant of preconception care provision. It specifically demonstrated the importance of various aspects of human capital, i.e., knowledge, perceptions, competence and adequacy of training in the provision of this care. Furthermore, it showed that the nursing cadre has a higher probability of providing this care.

### Study Limitations

The study utilized self-reported information which could have introduced social desirability bias. To minimise this bias, we used self-administered questionnaires to collect information from the respondents because it reduced the salience of social cues by isolating the subject. Further, we used a minimum sample size which could have resulted in insignificant results where there could have been an effect.

### Recommendations

We recommend holding frequent on job training and updates to reposition PCC as an important service and to enhance the knowledge and perceptions of health care providers especially nurses on PCC. This may contribute to an increase PCC engagement. Development of manuals and protocols to act as quick daily reminders, checklists during sessions especially in the rural and primary level facilities could also be valuable. Establishing a reporting system for PCC activities, and providing care in primary health facilities in rural areas can improve PCC service delivery. Community based studies should be conducted to further profile determinants of PCC provision and uptake in both rural and urban populations.

### Ethics approval and consent to participate

Ethical review was done by the MMUST Ethical Review Board (MMU/COR;403012 vol 2(5) and Jaramogi Oginga Odinga Teaching and Referral Hospital (ERC. IB/vol.1/448). The permission to conduct the study was given by the National Commission for Science, Technology and Innovation (NACOSTI) license number NACOSTI/P/18/22295/24670. During the study, informed consent was sought with full information being provided and comprehension being affirmed. Confidentiality was ensured through anonymity (using unique numbers), privacy during interviews and withdrawal at any point. For further inquiry into the research, the respondents were provided with the contacts of the principal investigator. The questionnaires were archived soon after data entry. During analysis, personal identifiable information was coded. The spreadsheet was password-protected and encrypted. Facility names and key informants' names were not used during reporting
